# Physical exploration of a virtual reality environment: Effects on spatiotemporal associative recognition of episodic memory

**DOI:** 10.3758/s13421-020-01024-6

**Published:** 2020-02-26

**Authors:** Daniël van Helvoort, Emil Stobbe, Richard Benning, Henry Otgaar, Vincent van de Ven

**Affiliations:** 1 Clinical Psychological Science, Faculty of Psychology and Neuroscience, Maastricht University, P.O. Box 616, Maastricht, 6200 MD the Netherlands; 2 Cognitive Neuroscience, Faculty of Psychology and Neuroscience, Maastricht University, P.O. Box 616, Maastricht, 6200 MD the Netherlands; 3 Leuven Institute of Criminology, Catholic University of Leuven, P.O. Box 3418, Leuven, 3000 Belgium

**Keywords:** Episodic memory, Associative recognition, Event segmentation, Interactive fidelity, Virtual reality

## Abstract

Associative memory has been increasingly investigated in immersive virtual reality (VR) environments, but conditions that enable physical exploration remain heavily under-investigated. To address this issue, we designed two museum rooms in VR throughout which participants could physically walk (i.e., high immersive and interactive fidelity). Participants were instructed to memorize all room details, which each contained nine paintings and two stone sculptures. On a subsequent old/new recognition task, we examined to what extent shared associated context (i.e., spatial boundaries, ordinal proximity) and physically travelled distance between paintings facilitated recognition of paintings from the museum rooms. Participants more often correctly recognized a sequentially probed old painting when the directly preceding painting was encoded within the same room or in a proximal position, relative to those encoded across rooms or in a distal position. A novel finding was that sequentially probed paintings from the same room were also recognized better when the physically travelled spatial or temporal distance between the probed paintings was shorter, as compared with longer distances. Taken together, our results in highly immersive VR support the notion that spatiotemporal context facilitates recognition of associated event content.

## Introduction

Although our senses are subjected to a continuous flow of information, memories of the past are retrieved as discrete episodes of perceptual and spatiotemporal information. That is, episodic memories consist of the “what,” “where,” and “when” elements of events (Mahr & Csibra, 2018; Tulving, 2002). During episodic memory formation, these elements are bound in an associative conjugative process, such that each element may form a retrieval cue for the entire memory. Thus, spatial and temporal context are core aspects of episodic memories (Eichenbaum, 2017; Howard & Eichenbaum, 2013; Miller et al., 2013; Moser, Kropff, & Moser, 2008; O’Keefe & Nadal, 1978; Ranganath, 2010; Tulving, 1993; Vargha-Khadem, 1997).

Whereas previous behavioral studies on how spatial context (e.g., navigation, orientation) facilitates episodic memory are relatively abundant, studies on how temporal context (e.g., order, duration, proximity) impacts episodic memory are relatively scarce. In addition, while some studies involve active navigation (e.g., motoric control), studies that have investigated these matters in ecologically rich settings involving free physical movement seem absent so far. To address these issues, we designed two museum rooms in virtual reality (VR) in which participants physically walked around freely while adhering to a pre-instructed route (i.e., no volition). On a subsequent old/new recognition task, we examined to what extent shared associated context (i.e., spatial boundaries, ordinal proximity) and the novel aspect of physical exploration of the environment (i.e., physically travelled spatial or temporal distance) facilitated recognition of museum room content.

Several theories postulate how continuous sensory input may be organized into discrete episodic memories. Event segmentation theory (EST) holds that while experiencing the world, adaptive prediction and planning of future experience and action is guided by a mental comparison of the mental representational properties of the ongoing event (i.e., “the working model”) against previously experienced events (i.e., “event models”). According to EST, episodic memories are segmented by “event boundaries” that occur when shifts in context give rise to prediction errors of near-future experience (Radvansky & Zacks, 2017; Richmond & Zacks, 2017; Zacks, Speer, Swallow, Braver, & Reynolds, 2007). Alternatively, context-retrieval models hold that items are bound to a context representation that gradually drifts over time, such that proximate items are indirectly linked by partial overlap in context (Howard & Kahana, 2002; Polyn & Cutler, 2017; Polyn, Norman, & Kahana, 2009). Taken together, episodic retrieval appears facilitated by shared associated context, yet disrupted by an abrupt shift or gradual drift in context (Dubrow & Davachi, 2013; DuBrow, Rouhani, Niv, & Norman, 2017).

Many behavioral studies support the notion that spatial context facilitates episodic memory. For instance, spatial navigation appears enhanced by associated topographical cues and disrupted by shifts in cue location (e.g., see the classic “Morris water maze” experiment (Morris, 1984), or for a review of similar studies in VR see Hamilton, Johnson, Redhead, & Verney, 2009). Other studies showed that orientation and self-motion facilitate episodic memory (e.g., see King, Burgess, Hartley, Vargha-Khadem, & O’Keefe, 2002; Simons & Wang, 1998). In contrast, behavioral studies on how temporal context may improve episodic memory are relatively scarce. Classic findings are that temporal order facilitates recall (Howard & Kahana, 1999, 2002) and recognition of items that are probed in the encoded order (Light & Schurr, 1973). In addition, explicit and implicit knowledge of temporal duration was found to facilitate memory processes via expectancy and enhanced sensory processing of target stimuli (e.g., van de Ven, Kochs, Smulders, & De Weerd, 2017; Vangkilde, Coull, & Bundesen, 2012). Further, behavioral studies provide evidence that not just temporal order, but rather ordinal proximity (i.e., temporal contiguity) may enhance recognition. This was first shown in a behavioral study by Schwartz, Howard, Jing, and Kahana (2005): In an old/new recognition task of previously shown images, successively probed old items were more often correctly recognized when the directly preceding item was encoded at a proximal position (relative ordinal position [lag] = -1 or +1) than at a distal position (lag >10, bidirectional). This effect was almost entirely attributable to cases in which the first image received a highest-confidence judgment (“sure old”).

Relevantly, other studies emphasized how shifts in associated context (e.g., “event boundaries”) may disrupt retrieval from long-term memory. Ezzyat and Davachi (2011) manipulated the suggested temporal proximity between a protagonist’s actions in a narrative (i.e., by insertion of the verbal temporal boundary “a while later”). The study revealed that participants’ memory for pre-boundary sentences was lower than for post-boundary or control (“a moment later”) pre- and post-boundary sentences. Likewise, in another study, memory for video narratives was impaired when event boundaries were removed (Schwan & Garsoffky, 2004). Similar effects on associative memory (i.e., on recency discrimination and temporal proximity judgments) were found in studies that used visual image sequences as the encoded stimuli (e.g., Dubrow & Davachi, 2013, 2016; Ezzyat & Davachi, 2014). Furthermore, recently one study showed that although perceptual event boundaries impaired associative memory for cross-boundary image pairs, associative memory was enhanced for single images that flanked the event boundaries (Heusser, Ezzyat, Shiff, & Davachi, 2018).

Only few studies have begun to assess how associated context may facilitate or disrupt long-term memory in settings that are more naturalistic than narrative and image-based studies. One VR study investigated the effect of spatial boundaries on order judgments for long-term episodic memory (Horner, Bisby, Wang, Bogus, & Burgess, 2016). Participants navigated (via keyboard and computer screen) a VR environment of 48 adjacent rooms that were separated by doors. Each room contained two objects for which participants had to identify whether they were man-made or natural. Subsequently, participants received a three-alternative forced-choice sequential memory task (“which object came before/after?”), in which half of the cue-target pairs were encountered in the same room and the other half in directly adjacent rooms. Results showed that temporal order judgment for two sequential objects was more accurate when both objects were encountered within the same room than between adjacent rooms, regardless of forward or backward temporal lag. This effect persisted when encoding time and spatial and temporal distance between objects were controlled for.

Brunec, Ozubko, Barense, and Moscovitch (2017) examined to what extent temporal duration and order judgments for event content depended on recollection or familiarity-based memory representations. Participants repeatedly viewed an automatically navigated route through traffic in a VR environment on a computer screen (derived from Google Streets View of Chicago). The route had stops of variable durations at intersections. Participants were instructed to remember as many details as possible about the route and its intersections. Thereafter, participants received an old/new recognition task containing 16 intersections and had to indicate for each whether they were recollected, familiar, or new. They were also shown intersections in pairs and in a list, which they had to sort on order and on temporal duration. Participants reliably indicated temporal duration for intersections that were recollected, but not for those that were familiar. In contrast, order judgments were accurate for both recollection- and familiarity-based judgments. Thus, the ability to recollect events seemed essential for temporal duration but not order judgments. In sum, these findings indicate that spatial and temporal contextual features are important in forming and retrieving perceptual events from memory.

Our current study uses a novel design to test contextual memory effects. That is, participants physically explored a naturalistic VR environment in which they were immersed in a physically unconstrained manner. Our study adds in several ways to the existing literature. First, neural substrates of memory in conventional lab studies appear to differ significantly from those for tasks in autobiographic environments (e.g., Cabeza et al., 2004; Chen, Gilmore, Nelson, & McDermott, 2017). Thus, conventional memory effects should be replicated in ecologically rich, naturalistic environments. VR may be a suitable candidate, as neural correlates activated by VR seem largely similar to those underlying real-life situations (e.g., see Aronov & Tank, 2014; Mellet et al., 2010; Plank, Snider, Kaestner, Halgren, & Poizner, 2015; but for differences, see: Aghajan et al., 2015). Moreover, information learned in VR transfers to, and can be reliably assessed in, real-life environments (Connors, Chrastil, Sánchez, & Merabet, 2014; Lloyd, Persaud, & Powell, 2009). Relevantly, our VR environment appears a suitable approach to an ecologically valid environment due to the high relative fidelity of sensory immersion (e.g., HD visuals, background sound, light conditions) in our VR design. Other issues relevant to the use of VR for psychology research in general are reviewed elsewhere (see Wilson & Soranzo, 2015).

Second, an important aspect of our VR design over conventional studies regards interactive fidelity (for a thorough review of the relevance of immersive and interactive fidelity and other challenges to the study of episodic memory in VR, see Smith, 2019). Some studies found that active navigation (keyboard-controlled) of a screen-based VR environment increased episodic memory relative to passive viewing (e.g., Hahm et al., 2007; Sauzéon et al., 2012; Sauzéon, N’Kaoua, Arvind Pala, Taillade, & Guitton, 2016). Other studies emphasized that interaction consists of two major components: “volition” (i.e., freedom of choice about how to interact with the environment) and “motoric control” (i.e., the act of physical interaction). Participants navigated through a VR city via a steering wheel and pedals. Here, motoric control impaired item recognition (but not spatial memory) compared to conditions of passive viewing, which in turn performed worse than the volitional control condition (see: Jebara, Orriols, Zaoui, & Berthoz, & Piolino, 2014; Plancher, Barra, Orriols, & Piolino, 2013). Relevantly, Laurent, Ensslin, and Marí-Beffa (2016) provided a critical note, in that intensive motoric control may have distracted attention from encoding. Finally, a recent study showed that the combination of both volitional and motoric control may have additive benefits (Chrastil & Warren, 2015). Overall, the effects of degree and type of control (motoric vs. volitional) still remain to be disentangled. Our current experiment is novel in that participants who immersed into our VR environment were allowed extensive motoric control (i.e., physical exploration). We did not involve conditions of passive viewing or volitional control.

Finally, our VR design benefitted not only ecological validity over conventional paradigms but also experimental control and means of data analysis. Beyond standardization and manipulation of stimuli, our paradigm allowed for the tracking and logging of physical movement of participants and their viewing direction and viewing time. This enabled the novel analysis of whether physically travelled spatial and temporal distance modulated subsequent memory performance, which provides crucial insights into how overt and physical interaction with a naturalistic environment affects our episodic memory of it.

In our study*,* we evaluated how context modulates recognition accuracy. Participants physically walked on a pre-instructed route (on which there were no physical boundaries to prevent them from wandering off) in two virtual museum rooms that each contained nine paintings and two stone sculptures. They later received an old/new recognition task for the paintings. We expected that participants would more often correctly recognize a sequentially probed old painting if the directly preceding painting was encoded within the same room than across rooms (e.g., see Horner et al., 2016), or in ordinal proximity rather than in ordinal distance within the same room (e.g., see Schwartz et al., 2005). Finally, in an exploratory analysis, we investigated whether physical exploration parameters, such as travelled distance between paintings in the same room, affected subsequent memory recognition. To this end, we developed a new analysis approach, which we term the distance-based mnemonic probability function.

## Method

All data and (non-copyright) material associated with this article are freely available on the Open Science Framework at https://osf.io/emk6d.

### Participants

Thirty students (*M*_age_**=** 21.7 years, *SD =* 2.3, range: 18–28 years, 20 women, 10 men) from Maastricht University were recruited via flyers and participated in the study. A post hoc power analysis revealed that with a sample size of 30, alpha set at .05, two-tailed, and a medium effect size (Cohen’s *d* = .5), a power of 0.75 was achieved for paired-samples t-tests (calculated via G*Power 3; Faul, Erdfelder, Lang, & Buchner, 2007). We screened participants so that only students who reported to have never experienced VR and had 100% normal or corrected vision entered the experiment. Participants were also screened via a “Yes/No” self-report questionnaire that consisted of a checklist of neurological, cardiovascular, and mental disorders, as these might be negatively affected by VR-induced “Simulator Sickness” (e.g., symptoms such as nausea, fatigue, headache, disorientation, arousal, and increased heart rate) that a minority of VR participants typically experience (Nichols & Patel, 2002; Sharples, Cobb, Moody, & Wilson, 2008). The study was approved by the Ethical Review Committee of the Faculty of Psychology and Neuroscience of Maastricht University, Maastricht, the Netherlands. Participation was awarded by financial compensation or course credit.

### Materials

#### VR lab, hardware and software

The experiment took place in a behavioral laboratory at Maastricht University designed for the purpose of VR research. The laboratory consisted of a control area for the researcher and a behavioral area of 4–6 m^2^ in which participants could physically freely walk around the VR simulation. In both areas, screens were located on which the researcher could monitor in 2D the content of the 3D VR simulation. The VR hardware consisted of a tracking system (http://www.phasespace.com/) and a head-mounted display (http://www.nvisinc.com/) that received the VR output through wireless high-definition multimedia interface (HDMI), giving the participant full freedom of physical movement to explore the VR environment. The visual simulation software Vizard 5 (http:/www.worldviz.com/) was employed to simulate the physical environment of two museum rooms. A script written in Python logged the participants’ XY position in the environment, including viewing direction and location sampled at 10 Hz (100 ms temporal resolution) and at millimeter resolution. The museum rooms and decorations were created in the 3D software Blender 3D (http://www.blender.org/).

#### Virtual museum rooms

**Fig. 1:**
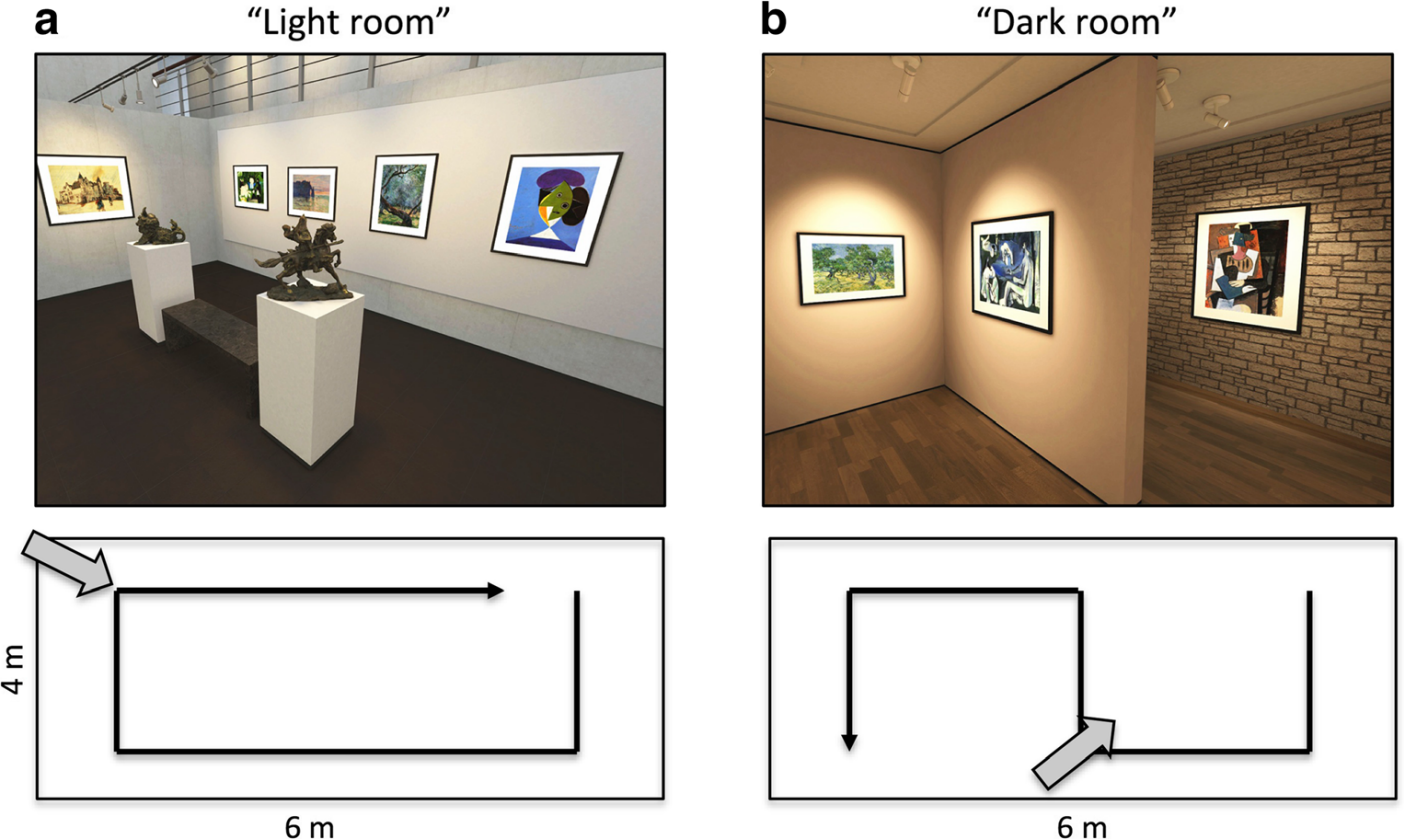
Virtual reality (VR) environments. Shown are print screens of the “light” (**A**) and “dark” (**B**) museum rooms in the VR simulation. Insets appended to each print screen show the general layout of the room and the instructed walking direction for exploration marked by the black arrow-headed path. The gray arrow indicates the approximate viewing direction of the print screens

Two museum rooms were designed to vary in spatial layout, decorative objects, light conditions, and sound. Both VR rooms spanned the full physical space of the behavioral area. One room was referred to as “the light room” (see Fig. 1A for a screenshot). The room had a modern design: concrete walls, a skylight ceiling, and spotlights. The room was constructed in an open rectangular shape. Participants could walk around a (virtual) marble bench and two bronze statues, which were located in the middle of the room. The walls contained nine framed paintings in total. The room was surrounded in 360° by sound that simulated background noise of museum visitors (e.g., whispering, footsteps). A second room was referred to as “the dark room” (see Fig. 1B for a screenshot). This room had a classic design, which involved dimmed light and warm tones of wallpaper. The room was constructed in a fixed S-shape, which obscured the participants’ view to most of the room’s content at any position. The walls contained nine framed paintings in total and two stone-carved sculptures. This room did not contain any background sound.

#### Stimuli

We used digitized samples of real paintings that were rendered and framed in 3D from high-definition images (*M*_resolution_ ± 1,600 × 1,600 pixels). We chose paintings as target items because they can be naturalistically rendered in 3D from their 2D counterparts and because they look very similar from any angle, ensuring consistent encoding among participants. In online databases, we searched for impressionist paintings by Monet, Van Gogh, and Guillaumin and expressionist paintings by Picasso. We selected paintings only if they appeared in series that contained paintings of close resemblance, so that we would also obtain “lures” for the recognition task. We obtained 54 such pairs of paintings. Next, we removed all pairs that we thought were well known to the general public or otherwise standing out. Our final selection of 18 painting pairs involved pairs by Monet (*n* = 7), Van Gogh (*n* = 5), Guillaumin (*n* = 2), and Picasso (*n* = 4). In a pilot study, we verified that none of the participants recognized the selected paintings from encounters prior to the study. We matched paintings across the two rooms based on painter, style, color pallet, and content. Paintings in both rooms depicted landscapes with trees, human-like figures, a human and/or flowers, water or a river, and windmills.

We verified that the pictures in the two rooms were comparable in terms of visual features. A previous study showed that beta and gamma parameters of a Weibull distribution effectively captured the visual appearance of natural images, and that these parameters strongly correlated with brain activity (Scholte, Ghebreab, Waldorp, Smeulders, & Lamme, 2009). We used this approach to estimate beta and gamma parameters from the paintings in our study and found no statistically significant difference between the two rooms (beta: *t*(16) = -0.29, *p* = 0.77; gamma: *t*(16) = -0.10, *p* = 0.91). Bayes factors showed that the data were more likely to occur under the null hypothesis of no difference for both parameters (beta: *BF*_*01*_ = 2.36; gamma: *BF*_*01*_ = 2.42). This indicated that the paintings were similar in visual content across the rooms.

#### Old/new recognition task

A recognition task was programmed in Psychopy 1.8 (Peirce, 2007). The recognition task consisted of 77 trials. Each trial prompted participants with a single painting. Fifty trials contained an “old” painting, i.e., a painting encoded in one of our simulated museum rooms. Specifically, the four “boundary” paintings (first and last paintings of the rooms) were prompted on two trials each, while the remaining 14 “old” paintings were prompted on three trials each. The outstanding 27 trails consisted of 18 “lure” paintings that closely resembled the old paintings, and nine “new” paintings that did not look like the old-lure painting pairs. The choice to prompt paintings multiple times, as well as the variation in number of prompts, was a compromise to increase the amount of “sequential trials” that would be available for analysis.

Per participant, the recognition task shuffled the trials in random order, after which a number of sequential trials were automatically reordered to display paintings that were encoded in close or far ordinal proximity (14 pairings on average) or that were encoded in the same room or not (28 pairings on average). Per trial, participants had an unlimited amount of time to respond via a keyboard to the question “Did you see this image in one of the rooms?” Yet, the paintings were only displayed for a maximum duration of 5 s on the screen. Paintings also disappeared from the screen in case a response was provided before this time lapsed. A response with the “H” key signified “Yes,” the “J” key signified “No.” In the latter case, the next trial started. In case of responding “Yes,” the instruction “Did you see the picture in the Dark room or the Light room?” was shown. Subjects could respond with the “H” key to indicate the dark room and with the “J” key to indicate the light room. In case participants indicated on a trial that they had seen the painting in a particular room, the final question of the trial was “How confident are you about this answer?” A response with the “H” key signified “Sure,” the “J” key signified “Pretty Confident,” the “K” key “Not so confident,” and the “L” key “Completely unsure.”

### Procedure

We first familiarized participants with the VR laboratory. Thereafter, participants were instructed that they were about to explore two museum rooms, a “light room” and a “dark room,” though not necessarily in that order. Participants were instructed to follow a specific route throughout the rooms, which was graphically depicted on an A4 sheet. The instructions emphasized that they were only allowed to walk by and view objects once. Participants were recommended to take their time to memorize objects in detail. As an incentive against poor effort, participants were informed that “the best participant” would receive an extra reward, i.e., chocolate bars worth 3 euros. Next, participants were equipped with the VR gear and located at the starting position. To familiarize participants to VR, they were first immersed into a space that consisted of a tiled floor. When the participants indicated that they were ready to explore the museum rooms, they were immersed in the light or dark room, which was selected based on randomization. After completion of that room, participants raised their hand, were guided back to the starting position, and immersed into the second museum room. After completion of both rooms, participants were seated behind a computer screen on which the recognition task was administered. Participants received the following written instructions: “You will see a number of images. For each image, you will have to indicate if you saw it before in one of the two rooms, and how confident you are about your decision. Specific instructions on how to do this are presented during the task. Each image will only be shown for a few seconds. However, you have unlimited time to respond. Only after your responses, the next image will appear. It is possible that images are shown multiple times during this recognition task.” After the recognition task, participants were asked if they had ever seen any of the paintings prior to the experiment, to gauge whether they had non-experimental knowledge of the stimuli.

At the end of the experiment, participants filled in the ITC – Sense of Presence Inventory (ITC-SOPI: Lessiter, Freeman, Keogh, & Davidoff, 2001), a measure of “presence” (i.e., the subjective sense that a subject was mentally transported to the virtual environment; sometimes described as the sense of “being there”) as well as negative VR effects (e.g., headache, nausea, eye strain, or other symptoms of simulator sickness or disorientation) that participants may experience during and after exposure to a visually displayed environment. There is evidence that presence and simulator sickness may be negatively related (for a review, see Weech, Kenny, & Barnett-Cowan, 2019). Participants rated a set of 38 questions on these topics on a five-point scale from “strongly disagree” (1) to “strongly agree” (5). The ITC-SOPI also includes questions on the level of prior knowledge and experience that participants had with 2D or 3D images, VR, and other visually displayed or simulated environments. The ITC-SOPI was administered strictly as a confound measure in relation to physical exploration. Finally, participants received their financial compensation and were debriefed.

### Statistical analysis

#### Conventional analysis of recognition performance

Paired-samples t-tests were employed (two-tailed). We first examined whether memory performance depended on spatial or temporal context during encoding. For this analysis, we focused only on hit rates (HR; proportion of correctly responding “old” to an item on the recognition task) because half of the participants saw four or fewer lure trials in the spatial or temporal pairings. To assess the effect of spatial boundaries, HRs for sequentially probed items from within a museum room were compared to those from across museum rooms. That is, of all pairs of sequentially probed items of which the first item was correctly recognized, we tested whether the HR of the second item was higher when that item came from the same room during encoding as the first item of the probed pair. We compared HRs for source attributions to chance level performance (chance proportion = .5) via a one-sample t-test. To test for ordinal proximity effects, HRs were compared for sequentially probed items that were encoded within the same room in ordinal proximity (lag -3 to +3) versus in ordinal distance (lag ≤ -4 or ≥ +4). That is, of all pairs of sequentially probed items of which the first item was correctly recognized, we tested whether HR of the second item was higher if that item followed the first item proximally rather than distally during encoding. This approach is comparable to one used in a previous study (Schwartz et al., 2005). Effect sizes are reported for significant results. For non-significant results, we calculated the Bayes factor to test the likelihood that the data resulted from the null hypothesis over the alternative hypothesis of a difference between conditions (that is, *BF*_*01*_), using JASP (JASP Team, 2018). We also examined participants’ confidence ratings. Finally, for exploratory purposes, we examined whether memory sensitivity (d’) and HR for old paintings and false-alarm rate (FAR) for lure paintings differed between items relevant to the light room compared to the dark room.

#### Effect of physical exploration on recognition

To investigate whether physical exploration had an influence on subsequent memory recognition, we developed a two-step analysis procedure in which we calculated distance-based mnemonic probability functions (d-MPFs). First, we selected sequentially probed old paintings that were encoded from the same room and of which participants correctly recognized the first painting of each pair (see also the previous analyses). For each pair of paintings, we calculated the travelled spatial and temporal distance between the respective paintings during encoding from the VR log files. Spatial distance was calculated as the cumulative distance travelled between the two paintings in meters (using Matlab’s *distance* function). Temporal distance was calculated as elapsed time while traversing between the central positions of the two paintings in seconds. A probability distribution histogram was then calculated for the spatial or the temporal distances using five bins of equal distances in, respectively, meters or seconds, collapsed across the two rooms. In the second analysis step, for each spatial or temporal bin, we calculated each participant’s HR of the *second* item of each pair of paintings, resulting in d-MPFs that captured the probability for correct recognition judgments as a function of spatial or temporal distance. The d-MPFs of all participants were subsequently analyzed using a permutation-based T-test (Ernst, 2004; Welch, 1990; Mielke Jr & Berry, 1994), which is robust against violations of normality and variance homogeneity. The permutation p-value (*p*_perm_; two-tailed, obtained from 2,000 permutations) represents the proportion of the surrogate T values that is higher than the T value from the observed data.

## Results

### Spatial context in recognition memory

**Fig. 2:**
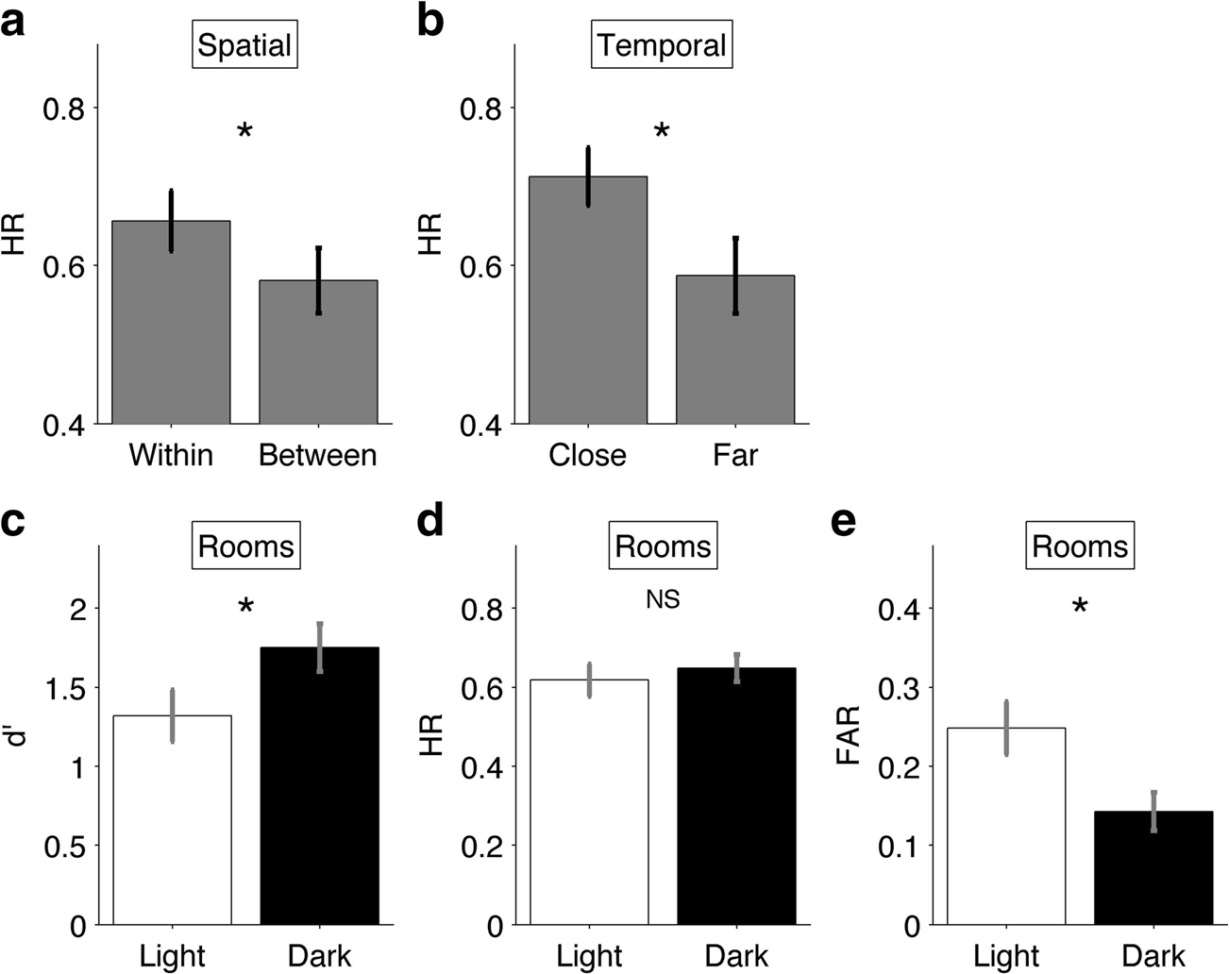
Results. Memory performance metrics for sequentially probed items coming from within the same or different rooms (**A**), for sequentially probed items encoded in proximal or distal ordinal distance (**B**), and for (**C**) d’, (**D**) hit rate (HR), and (**E**) false alarm rate (FAR) for the light and dark room. Error bars = SEM; * = significant difference (p < .05) between the two bars; NS = no significant difference

We examined to what extent shared associated context in the form of spatial boundaries influenced participants’ recognition performance. Sequentially probed old paintings on the recognition task from within the same museum room received a higher HR (*M =* .65, *SEM =* .04, *CI*_*95*_ = [.57, .72]) than those from across museum rooms (*M =* .58, *SEM =* .04, *CI*_*95*_ = [.50, .65]; Fig. 2A). This difference was statistically significant, *t*(29) = 2.14, *p* = 0.04, *d* = 0.36). Thus, sequentially probed paintings from within a museum room were more often correctly recognized than those from across museum rooms. We also ran an analysis of source attribution. The mean source HR (i.e., rate of correct source room attributions for recognized old paintings) for the light room (*M =* .74, *SEM =* .04, *CI*_*95*_ = [.66, .81]) was lower than that for the dark room (*M =* .81, *SEM =* .03, *CI*_*95*_ = [.76, .87]). This difference was not statistically significant, *t*(29) = -1.71, *p* = 0.09, but the evidence in favor of the null hypothesis was weak, *BF*_*01*_ = 0.71.

### Temporal context in recognition memory

Next, we examined whether sequentially probed old paintings from within the same room were more likely to be correctly recognized when encoded in ordinal proximity (lag -3 to +3; close pairs) compared to ordinal distance (lag ≤ -4 or ≥ +4; remote pairs). Participants’ mean HR for close pairs (*M* = .71; *SEM* = .04, *CI*_*95*_ = [.64, .78]) was higher than for remote pairs (*M =* .58; *SEM* = .05, *CI*_*95*_ = [.49, .68]; Fig. 2B). This difference was statistically significant, *t*(29) = 2.45, *p* = 0.02, *d* = 0.41. Thus, sequentially probed old paintings from within the same room were more likely to be correctly recognized when encoded in close ordinal proximity than in ordinal distance. Furthermore, the mean confidence rating for close pairs (*M* = 1.87, *SEM* = 0.12, *CI*_*95*_ = [1.63, 2.10]) was not statistically significantly different from the mean confidence rating for remote pairs (*M* = 1.77, *SEM* = 0.14, *CI*_*95*_ = [1.50, 2.04]; *t*(27) = 1.31, *p* = 0.20; *BF*_*01*_ = 2.30). Note that in this analysis of confidence ratings two subjects were excluded because of too few hits for one or more conditions.

To assess the directionality of the temporal lag effect, we split the temporal context analysis in forward and backward direction of ordinal proximity: By “forward temporal lag” we refer to items that were sequentially probed in the temporal direction of encoding, whereas by “backward temporal lag” we refer to items that were sequentially probed in the order opposite to their encounter in VR. As a follow-up of the general temporal lag effect, we conducted one-tailed t-tests for the forward- and backward-lagged analyses. We found a statistically significant effect of close versus remote pairs for backward-lagged sequentially probed paintings, *t*(29) = 2.16, *p* = 0.02, *d* = 0.37, such that the mean HR for close pairs (*M* = .68; *SEM* = .05, *CI*_*95*_ = [.58, .77]) was higher than the mean HR for remote pairs (*M* = .50; *SEM* = .07, *CI*_*95*_ = [.36, .63]). Participants’ recognition performance for item pairs in the forward-lag direction showed a similar trend, where close pairs had a higher mean HR (*M* = .77; *SEM* = .05, *CI*_*95*_ = [.68, .86]) than remote pairs (*M* = .66; *SEM* = .06, *CI*_*95*_ = [.55, .77]). This difference was not statistically significant, *t*(29) = 1.57, *p* = 0.06, but the effect size was moderately strong, *d* = 0.27.

### VR physically travelled distance and recognition memory

**Fig. 3:**
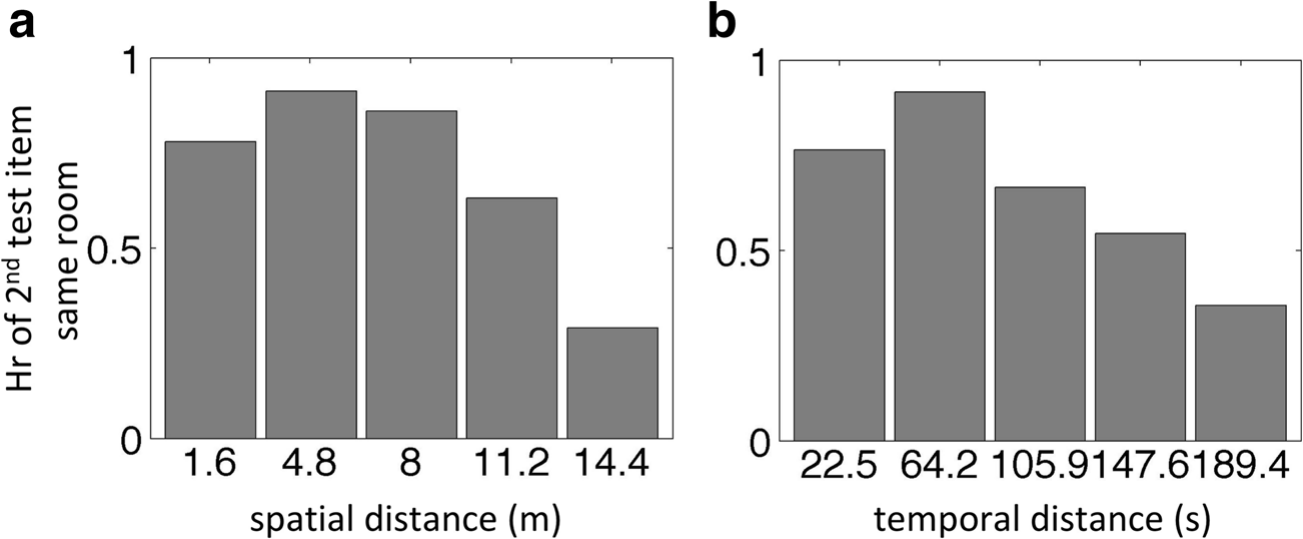
Virtual reality (VR) physical exploration (i.e., travelled spatial and temporal distance) effect on memory recognition. Bar plots show distance-based mnemonic probability functions (d-MPFs) for (**A**) travelled spatial distance (in meters, m) and (**B**) temporal distance (in seconds, s). Y-axis displays hit rates (HR) for the second item in a pair of sequentially tested items that were encoded in the same room, averaged across participants

To investigate whether physical exploration of the VR explained subsequent memory recognition, we analyzed the log files of the movements and viewing direction of each participant in the VR environment over time. Log files of three participants were missing and one file was corrupted. We calculated distance-based mnemonic probability functions (d-MPFs) for individually travelled spatial and temporal distances between picture pairs during encoding. As was to be expected, the spatial and temporal distances were highly correlated (*r* = .90, *p* < 0.001). Results showed that, pooled across both rooms, travelled spatial and temporal distance between paintings significantly predicted memory performance (spatial: *T*[18] = -4.1, *p*_perm_ < 0.001, Cohen’s d = -0.93; temporal: *T*[14] = -3.5, *p*_perm_ < 0.01, Cohen’s d = -0.91), with better recognition for shorter spatial or temporal distances compared to longer distances (see Fig. 3). Thus, VR exploration parameters modulated subsequent memory performance in either room.

### ITC-SOPI

We calculated average composite scores for each of the four subscales of the ITC-SOPI (mean (SD) of Spatial Presence = 3.51 (0.62); Engagement = 3.68 (0.49); Ecological Validity = 3.79 (0.69); Negative Effects = 2.54 (0.80)). None of the subscales correlated with performance on the memory recognition task (all *p*s > 0.13) or confidence ratings (all *p*s > 0.7). We also correlated memory performance and confidence ratings with “Motion Sickness” (items B14, B26 and B37 from the Negative Effects subscale; mean (SD) = 2.56 (1.06)), and found no significant effects (memory performance, p = 0.37; confidence ratings, p = 0.86). From this we conclude that negative side effects of VR did not affect subsequent memory performance.

### Exploratory analysis of cross-room effects

**Fig. 4:**
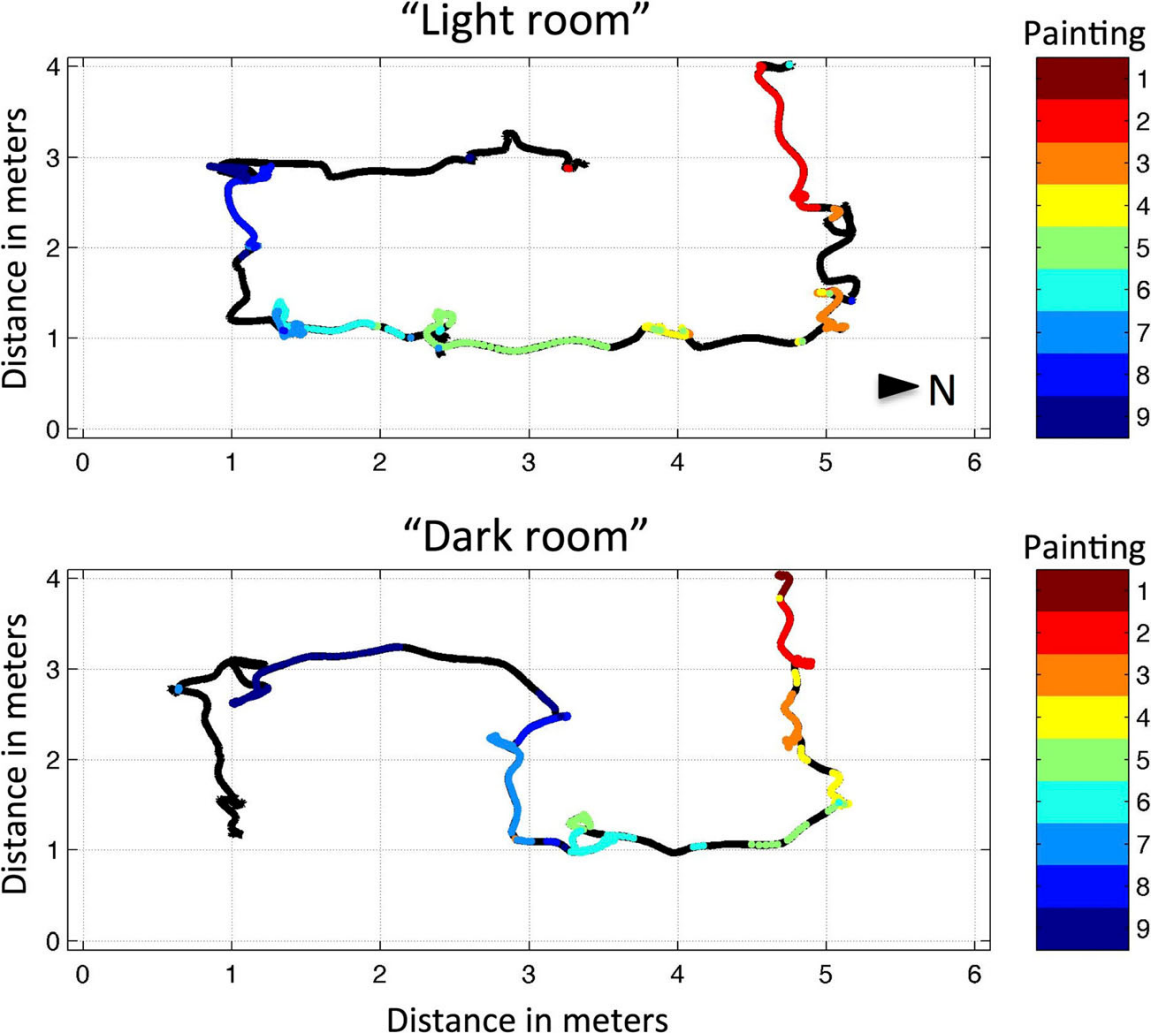
Museum path trace of a selected participant. The trace (black line) marks the physical locations that the participant visited within the virtual environment, with XY coordinates in meters. Colors indicate moments along the track at which virtual reality (VR) helmet gaze was directed to each of the virtual locations of the nine paintings (see color legend). Trace information can be used to calculate travel and viewing times and visited locations in the VR environment. Compare paths to insets of Fig. 1. Arrow head indicates North

For exploratory purposes, we analyzed recognition effects across rooms. Figure 4 depicts the route taken in each room by a randomly selected participant. Based on the log files, we found that participants on average spent a comparable amount of time in the light room (*M* = 285.30 s, *SEM* = 33.21, *CI*_*95*_ = [220.21, 350.39]) to in the dark room (*M* = 290.80 s, *SEM* = 21.35, *CI*_*95*_ = [248.95, 332.65]; *t*(25) = -0.27, *p* = 0.79). Moreover, viewing time of each of the paintings was similar between the light room (*M* = 20.42 s, *SEM* = 2.72 s, *CI*_*95*_ = [14.82, 25.46]) and the dark room (*M =* 19.38 s, *SEM* = 1.63 s, *CI*_*95*_ = [16.18, 22.59]), *t*(25) = 0.50, *p* = 0.62.

Participants’ mean *d’* differed statistically significantly (*t*(29) = 2.10, *p* = 0.045, *d* = 0.38) between the light (*M* = 1.32, *SEM* = .16, *CI*_*95*_ = [.98, 1.66]) and the dark room (*M* = 1.75, *SEM* = .15, *CI*_*95*_ = [1.44, 2.06]), suggesting that recognition memory was better for the dark than for the light room (Fig. 2C). To further investigate this effect, we analyzed the HR and FAR. Mean HR for the light room (*M =* .62, *SEM =* .04, *CI*_*95*_ = [.54, .70]) was not statistically significantly different from that of the dark room (*M =* .65, *SEM =* .03, *CI*_*95*_ = [.58, .72]; *t*(29) = -0.72 *p* = 0.48; Fig. 2D). The Bayes factor further indicated that the data were four times as likely to result from the null hypothesis of no difference (*BF*_*01*_ = 4.06). Mean FAR differed significantly (*t*(29) = -3.14, *p* = 0.004, *d* = -0.57) between the light (*M* = .25, *SEM* = .03, *CI*_*95*_ = [.18, .32]) and the dark room (*M* = .14, *SEM* = .02, *CI*_*95*_ = [.09, .19]), suggesting that participants tended to falsely recognize lures when they resembled items from the light room as compared with the dark room (Fig. 2E). Participants’ mean confidence rating for hits regarding the light room (*M* = 1.80, *SEM* = 0.11, *CI*_*95*_ = [1.58, 2.01]) was not statistically significantly different from their mean confidence rating for hits concerning the dark room (*M* = 1.86, *SEM* = 0.10, *CI*_*95*_ = [1.66, 2.05]; *t*(29) = -0.26, *p* = 0.80; *BF*_*01*_ = 4.98).

## Discussion

Previous behavioral studies on how context facilitates episodic memory recognition in humans typically addressed the matter for encoded narratives, (motion) pictures, or screen-based VR (e.g., see Burgess, Maguire, & O’Keefe, 2002; Horner et al., 2016). To our knowledge, the present study was the first to examine this issue via a VR environment in which participants could physically explore the environment, and to subsequently analyze effects of physically travelled spatial or temporal distance on associative memory.

Our main findings on the old/new recognition task can be catalogued as follows. First, we found that a sequentially probed old painting was more often correctly recognized when the directly preceding painting was encoded within the same VR museum room than across rooms (a “spatial boundary effect”). Second, sequentially probed old paintings were more often correctly recognized when the directly preceding painting was encoded in close ordinal proximity than in relative ordinal distance (within a room; an “ordinal proximity effect”). Finally, our most important and novel analyses showed that the “physically roamed spatial and temporal distance” between paintings was associated with the spatial and temporal context effects on sequentially probed paintings that were encoded within the same room. To our knowledge, we are the first to show that physically travelled distances during exploration affects subsequent memory performance. These findings support the notion that spatial and temporal contextual features are important in forming and retrieving perceptual events from memory.

The “spatial boundary effect” that we observed in our VR paradigm is similar to findings by previous screen-based VR studies that required participants to navigate a series of rooms. In one study, participants whose avatar carried objects in a VR room had more accurate short-term memory for carried objects if they were tested within the same room than if they were tested in another room and/or had returned to the original room (Radvansky & Copeland, 2006). Similarly, Horner et al. (2016) showed that long-term order memory for objects on a forced-choice recognition task was better for cue-target objects from within the same room than for cue-target pairs from directly adjacent rooms. Contrary to Horner and colleagues, we found that source monitoring (i.e., correct source room attribution for recognized old items) was higher than chance level, although monitoring did not differ between the two rooms.

The “ordinal proximity effect” in our study adds to the few behavioral studies that have investigated how episodic memory is facilitated by temporal context. Our finding in physically explored VR closely resembles that of Schwartz et al. (2005), who showed that recognition of the second image of sequentially probed old images was enhanced when these pairs were encoded in relative ordinal proximity (i.e., close forward or backward relative ordinal position [lag]) than ordinal distance. In their study, this associative effect appeared almost wholly attributable to cases in which the first item of a successive old pair on the recognition task received a highest-confidence judgment (“sure old”). Although this associative effect of lag was bi-directional, when we split our own data into item pairs that were forward- and backward-lagged, only the backward-lag effect was statistically significant. However, as the forward-lag data in our study represented a nonsignificant moderate effect of the same trend, we speculate that this effect was not significant, perhaps due to our limited sample size.

Theories that hold that episodic memories are bound by context and catalogued either by abrupt shifts or gradual drifts in context over time are consistent with our findings. Specifically, the “spatial boundary effect” fits with event segmentation theories that suggest that episodic memories are segmented by context shifts that lead to prediction errors of near-future experience (e.g., Radvansky & Zacks, 2017; Zacks et al., 2007). In addition, the “ordinal proximity effect” supports temporal- and context-retrieval models that hold that context representations gradually drift over time, linking proximate items via partial overlap in context (e.g., Polyn & Cutler, 2017; Polyn et al., 2009). Admittedly, these findings also neatly fit neuroscience studies that find that spatiotemporal context central to episodic memory formation and retrieval involves specialized neural cells (i.e., place, grid, orientation, and time cells) in the hippocampus (e.g., see Eichenbaum, 2017; Howard & Eichenbaum, 2013; Miller et al., 2013).

In an explorative analysis, we found that the FAR was lower for items from the “dark” room than from the “light” room. One interpretation is that this finding might be attributable to geometric differences between the rooms. It is possible that the dark room may contain more event markers (Heusser et al., 2018; Horner et al., 2016) due to local geometry (i.e., S-shape, narrow alley, corners), and that its restricted view may enhance item-specific encoding, decreasing FAR (Hege & Dodson, 2004; McCabe, Presmanes, Robertson, & Smith, 2004). In contrast, the light room contains open geometry, which may stimulate collective encoding, increasing FAR. However, as we did not counterbalance paintings across rooms – this was impractical at time of testing due to technical limitations – an alternative interpretation is that the effect is confounded by stimuli differences. Still, in considering alternative explanations, we found no differences across rooms based on the visual features (beta and gamma parameters) and viewing time of paintings. Although this suggests a context effect, our design prevented us from further investigating this issue in detail. However, we consider this finding a proof of principle that is important to examine in a follow-up study.

Some limitations of the present study deserve comment. First, we did not include a control group of passive viewing, or a condition in which participants were given volitional control. Future studies could include a (screen-based) VR group in which a prerecorded 3D video of the VR environment is passively viewed (e.g., based on the log files of exploring participants). Alternatively, control groups could involve participants moving the environment via keyboard. Such approaches would enable direct data comparisons across screen-based and physical-movement based VR groups. In addition, future studies may opt to allow participants to determine their itinerary prior to physically exploring the rooms, or “on the go” rather than to instruct them to follow a specific route. Volition may increase the degree of interaction and enhance episodic memory (see Jebara et al., 2014). Further, technical limitations at the time of testing involved that online randomization (e.g., counterbalancing) of paintings would lead to novel and delayed rendering (because of differences in frame sizes of the paintings, resolution, light settings, etc.). This would have caused pauses or breaks between the virtual rooms that we deemed impractical and distracting for participants. Hence, we opted to match paintings across rooms rather than to counterbalance them. As a result, any analyses across rooms remained exploratory. Current software and technology in our lab do not suffer from these limitations anymore. Finally, to acquire sufficient “sequential pairs” for data analysis, we opted for the compromise to prompt old paintings multiple times. This could lead to “memory updating,” although participants did not receive feedback on their responses.

A relevant note regarding our analysis of physically travelled distance effects is that previous studies have shown that distance estimation in VR appears to be somewhat compressed compared to real-life physical distances (Lampton, McDonald, Singer, & Bliss, 1995; Renner, Velichkovsky, & Helmert, 2013; Witmer & Kline, 1998). This may in part be due to VR hardware and technology (e.g., reviewed by Renner et al., 2013), with larger VR distance compression observed when using computer monitors compared to head-mounted displays (HMDs) comparable to those used in our study (Lampton et al., 1995; Willemsen & Gooch, 2002). Notably, our study focused on relative distances between items, thereby limiting a possible effect of VR distance compression on memory. However, it remains to be investigated how real versus VR environments may affect memory performance.

Inherent in our VR design are several strengths that make our paradigm worthwhile to use for future research on associative memory beyond effects of physical exploration. First, our stimuli can easily be standardized and manipulated (e.g., randomized, counterbalanced). Second, due to the flat surface of the stimuli, encoding is likely consistent across participants, regardless of viewing angle towards the paintings. This enhances validity and ease of use in recognition tasks. Third, in future studies, we can extend physical space using wire-free VR technology or virtual space (e.g., doors or “virtual teleportation” to separate spaces). This way, large sets of stimuli and large environments could be explored. Fourth, log files allow tracking of orientation, viewing time, and participant location, which enables analysis of interaction and roaming effects. Finally, we found no evidence for individual differences in negative effects of our VR setup, as measured by the ITC-SOPI, affecting memory performance.

Our paradigm lends itself well to investigate other important topics in associative memory research. First, as our stimuli consist of “series” of paintings, they can be used for a variety of false-memory studies in which “the twin paintings” are used as lures. Second, our paradigm could be used to examine how context influences order and/or temporal duration judgments while “free-roaming.” Current studies on this topic are screen-based (e.g. Brunec et al., 2017; Horner et al., 2016). Third, follow-up studies could include conditions of passive viewing and volitional control to investigate how aspects of interactivity other than physical movement may affect episodic memory (e.g., see Jebara et al., 2014; Plancher et al., 2013). Finally, in follow-up studies, other physical exploration effects such as incidental experiences or geometry effects may be isolated and investigated. We found that travelled spatial and temporal distances were strongly correlated and had similar effect sizes in explaining recognition performance.

## Conclusion

In a novel VR paradigm in which participants could physically explore the environment, we examined how context related to episodic memory recognition. Specifically, we showed that when items shared context in the form of spatial boundaries or close ordinal proximity, recognition performance was enhanced when such items were sequentially probed on an old/new recognition task. We also showed that physically travelled distances were associated with subsequent memory performance, thereby demonstrating the unique opportunities that physical exploration in VR may provide in studying memory in natural settings. These findings are consistent with theories that hold that episodic memories are catalogued by abrupt context shifts and/or gradual context drifts. Our VR paradigm is further applicable to a variety of episodic memory studies, e.g., on memory distortion effects, context effects on order and duration judgments, effects of interaction versus passive viewing, and the effect of geometric, environmental and incidental factors on episodic memory formation.
